# An in silico analysis of heart rate impact on wall shear stress hemodynamic parameters in aortic coarctation

**DOI:** 10.1038/s41598-025-85522-0

**Published:** 2025-01-22

**Authors:** Jie Wang, Emily Manchester, Alex Skillen, Malebogo Ngoepe, Bernard Keavney, Alistair Revell

**Affiliations:** 1 School of Engineering, The University of Manchester, Manchester, UK; 2 Division of Cardiovascular Medicine, The University of Manchester, Manchester, UK; 3 Centre for Research in Computational and Applied Mechanics, University of Cape Town, Cape Town, South Africa

**Keywords:** Coarctation of aorta (CoA), Hemodynamics, Computational fluid dynamics (CFD), Turbulent wall shear stress, Heart rate, Large Eddy simulation (LES), Biomedical engineering, Aortic diseases

## Abstract

This study examines how heart rate (HR) affects hemodynamics in a South African infant with Coarctation of the Aorta. Computed tomography angiography segments aortic coarctation anatomy; Doppler echocardiography derives inlet flow waveforms. Simulations occur at 100, 120, and 160 beats per minute, representing reduced, resting, and elevated HR levels. Turbulence was analyzed over time and space using turbulence-resolving and pulsatile large-eddy simulations. Specifically, a 60% reduction in HR led to a reduction in maximum velocity by 45%, and a 57% decrease in pressure drop. The reduction in turbulence-related metrics was less significant. The ratio of turbulent kinetic energy to total kinetic energy decreased by 2%, while turbulent wall shear stress decreased by 3%. These results demonstrate that HR significantly affects velocity and pressure drop, while turbulence arising from the coarctation region is relatively unaffected. The balance between turbulent kinetic energy and total kinetic energy shows minimal enhancement due to the complex interplay among HR, turbulence, and geometry. This complexity prompts discussion on how HR-slowing medications, such as beta-blockers or ivabradine, could positively influence hemodynamic stresses. In particular, the results indicate that while HR modulation can influence flow dynamics, it may not significantly reduce turbulence-induced shear stresses within the coarctation zone. Therefore, further investigation is necessary to understand the potential impact of HR modulation in the management of CoA, and whether interventions targeting the anatomical correction of the coarctation may be more effective in improving hemodynamic outcomes.

## Introduction

Congenital heart disease (CHD) is a significant global health concern, affecting 9 out of every 1,000 live births and contributing to a substantial burden of disease. While CHD is a leading cause of childhood mortality, accurate epidemiological data from Africa and low-income countries is scarce. Recent global analyses indicate a significantly lower prevalence of CHD in Africa compared to other regions, but this probably reflects a lack of up-to-date research rather than a true lower prevalence^1^. Aortic coarctation (CoA), characterised by narrowing of the aorta, is responsible for approximately 6-8% of live births with CHD and the estimated incidence of CoA is approximately 1 in 2500 births^2,3^. The prevalence of CoA was significantly lower in the cohort in southern Africa compared to the global prevalence study. CoA is a complex disease with large variability from patient to patient. In less severe cases, CoA shows a slight narrowing, while in extreme cases it shows a severe constriction, such as tubular hypoplasia^4,5^. The exact causes of CoA are not fully understood, and the fluid-associated phenotype may play an important genetic role in the initiation and development of cardiovascular disease^5–7^. Long-term studies have shown that patients who underwent surgical repair as children were at increased risk of heart problems throughout their lives. Research also suggests that abnormal blood flow, as well as the shape and size of the aorta, could explain the different types of CoA seen in patients^8–10^. More research is essential to deepen our understanding of the hemodynamics associated with CoA to understand its role in the pathophysiological processes of growth, remodelling, and development in disease progression^11,12^.

Various cardiovascular diseases, including CoA, produce an abnormal hemodynamic environment compared to the healthy cardiovascular system. CoA can produce a high velocity jet that can exhibit transitional and turbulent features over a cardiac cycle^13^. Turbulence is known to significantly affect the function of endothelial cells, which line the inner walls of the arterial wall^14–16^. In contrast to laminar flow, where endothelial cells align with blood flow, turbulent flow patterns disrupt alignment^17^. This disruption leads to an irregular shear stress environment, prompting endothelial cells to produce proteins that contribute to atherosclerotic processes.

Cardiovascular hemodynamic research can benefit from computational fluid dynamics (CFD) and medical imaging to create detailed models of blood flow within patient-specific vascular structures. These models improve our understanding of flow dynamics, providing critical information on cardiovascular health and disease. One of the central challenges in this domain is the accurate numerical modelling of blood flow turbulence, especially in cases of vascular stenosis. Initial research efforts have largely employed time-averaged solutions from Reynolds-averaged Navier–Stokes (RANS) equations^18–20^. Although RANS models can predict average flow characteristics under certain conditions, they struggle with complex time-varying flow dynamics, such as pulsatile transitional flows in stenotic regions. For a detailed portrayal of turbulence, scale-resolving techniques are necessary. Direct numerical simulation (DNS) offers complete resolution of turbulence features but at high computational cost. Large eddy simulation (LES) lies somewhere in between and acts to resolve the large energy containing eddies whilst modelling smaller eddies. LES offers a more cost-effective solution than DNS while capturing essential flow dynamics^20,21^.

Recently, LES has been used effectively to assess turbulence in realistic cardiovascular flow applications. Specifically, researchers have combined medical imaging with LES to create patient-specific models of healthy and diseased aortas^22–31^. Pioneering work by Lantz et al.^14^ investigated turbulence in realistic aorta models using magnetic resonance images (MRI) of healthy aortas in conjunction with LES. They implemented a Reynolds-like decomposition to support qualitative and quantitative investigations of the characteristics of wall shear stress (WSS)^13,14^. This methodology was extended to CoA patients, focusing on TKE analysis^13,32^. An inverse correlation was identified between the severity of the stenosis and TKE: As the dilation of the stenosis increased, the TKE decreased but was not completely eliminated^19^. The geometric influence on turbulence levels and flow eccentricity was also investigated^19,33^. Although patient-specific CoA LES studies are limited, Goodarzi-Ardakani et al. examined a standard geometry that represents patients with CoA and repaired CoA. They found minimal changes in the impact of aortic morphology on key hemodynamic indices^34^.

High-fidelity CFD allows for turbulent-related studies in arterial hemodynamic research. However, these models often rely on idealised properties under simplified conditions, such as fixed HR, stroke volume, blood pressure, geometry, and flow rate separation (the distribution of blood flow between upper and lower limbs). In reality, the cardiovascular system operates under dynamic conditions, and HR can vary significantly due to physiological or pharmacological factors. Researchers have shown that observation of hemodynamic variability over extended periods, rather than relying on time-averaged quantities, can significantly improve understanding of the dynamic roles of blood flow, shear stress, and arterial pressure. This approach allows for the detection of transitional phenomena that influence arterial function, diameter, and wall thickness, providing a more comprehensive view of cardiovascular health^35–37^. Existing numerical studies that evaluate turbulence in adult patients with CoA^19,38–40^. These studies either use inlet velocity profiles derived from medical imaging or literature. Typically, these are acquired *at rest*, meaning all present studies are representative of rested states. HR is highly variable, and the effect of HR on turbulence-hemodynamicss is yet unknown. This is of particular interest in cardiovascular diseases, such as CoA, where medications are intentionally used to lower HR. Beta-blockers lower HR and aortic pressure to manage pre- and post- operative hypertension in CoA patients. Directly addressing aortic obstruction through surgery is generally preferred, though in some cases, the procedure can be delayed. Surgery for stable young CoA patients can be delayed for growth, high surgical risk, or misdiagnosis^41^. In such cases, beta-blockers and other medications are used to manage symptoms and control hypertension until surgery can be performed safely^42^. In pediatrics, the use of beta-blockers is even more complex; children with CoA may experience varying effects compared to adults, and there are insufficient data to definitively support or oppose the use of beta-blockers in children with CHD^43^. The debate about its safety and effectiveness in this population is ongoing, as it can impact flow, structure, and CoA hemodynamics^10^, raising questions about how HR changes affect blood flow dynamics and the arterial wall. Therefore, the primary objective of this study is to investigate how different HR affects blood flow and related hemodynamic parameters in the aorta of an infant with CoA, particularly with respect to turbulence and related WSS markers. The use of Large Eddy Simulation allows for a more accurate accounting of the impact of HR on hemodynamics, which is essential to inform clinical decisions about HR-modulating medications such as beta-blockers.

## Results

### Flow characteristics

**Fig. 1:**
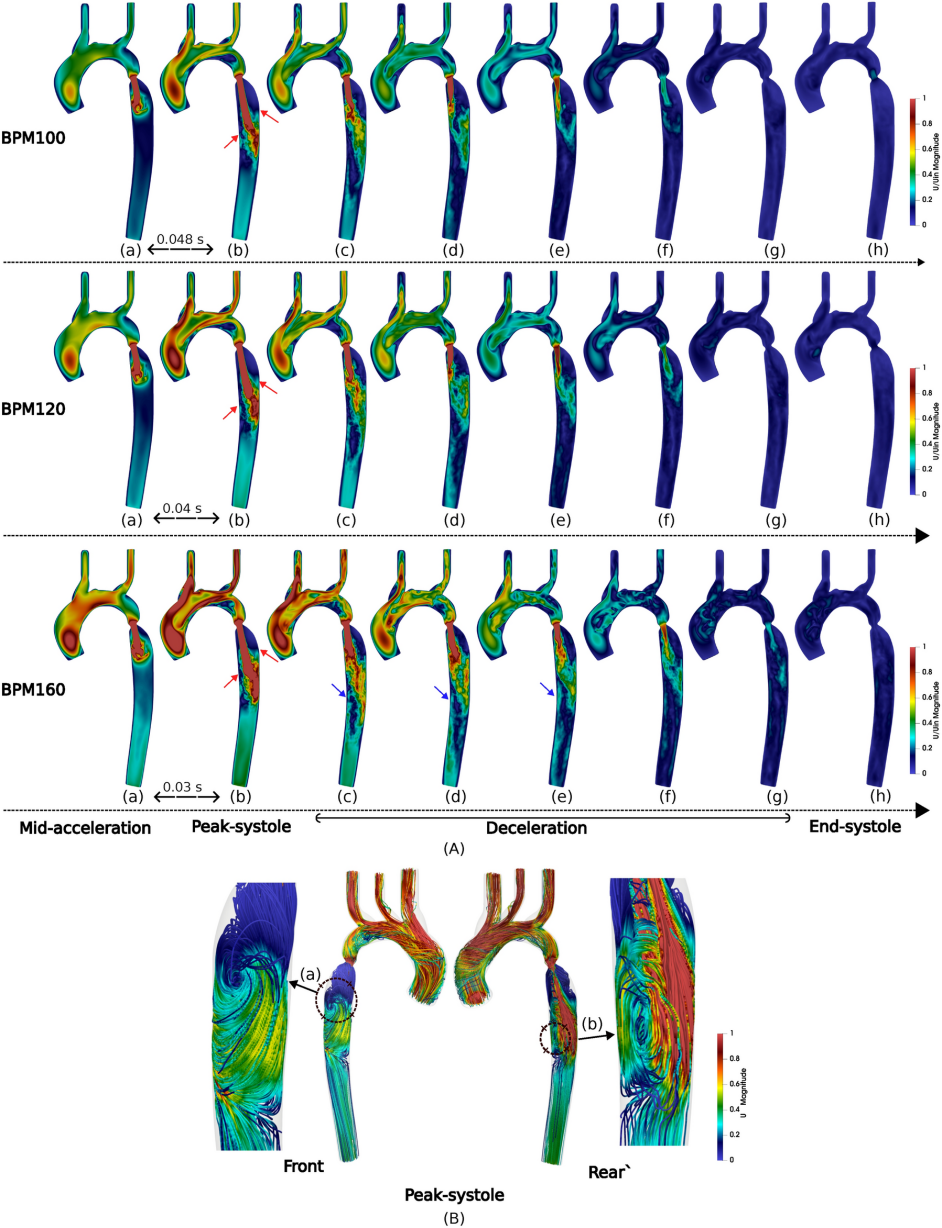
Flow velocity contours (**A**) are shown during mid-acceleration, peak systole, deceleration, and diastole. These contours are for three HR scenarios, colour-coded by the normalised maximum inlet velocity of BPM120. Note that the parabolic inlet profile is not visible due to clipping. Arrows mark areas of interest. Velocity streamlines show flow recirculation regions at peak systole for 120 BPM (**B**).

The resulting flow characteristics for each HR are presented in Fig. 1, illustrated with eight snapshots of the velocity contour during different phases of the cardiac cycle, namely mid-acceleration, peak systole, deceleration and end systole. These snapshots collectively highlight the evolution of blood flow through various stages of the cardiac cycle, showcasing the changes in jet flow velocity and pattern over time. In all cases, a high-velocity jet develops in the coarctation region throughout the systolic acceleration, reaching maximum intensity at peak systole. The jet breaks down through systolic deceleration and diastole. Similar behaviours have been well captured and described in several CoA studies^14,32,44,45^. In the present work, the observed jet flow exhibits an oscillatory motion in the anterior-posterior direction downstream of the constriction. This oscillatory motion is caused by the presence of eccentric or asymmetric stenosis, where the constriction is not aligned with the central axis of the vessel. Furthermore, diastole and subsequent deceleration have a destabilising effect on the jet, and this oscillatory behaviour is observed during systolic deceleration. At increased HR, the jet extends further downstream of the coarctation, the flow covers a greater distance and area, leading to increased interaction with the vessel wall in the distal portion of the descending aorta, as shown in the case of BPM160. In all three cases, the same oscillatory behaviour is observed, but in BPM160 there is a time delay of 0.015 s compared to BPM100 and 120. This is probably due to the higher energy flow in BPM160, which helps to maintain a stable jet flow for longer during deceleration. Flow visualisations of the recirculating flow are provided in Fig. 1B, where indicated recirculation regions are observed to remain in roughly the same region regardless of HR and are highlighted by the red arrow in Fig. 1A. The presence of recirculation is consistent with observations made in a retrograde flow study conducted by Fuchs et al., where normal laminar flow can be disrupted and cause regions of flow reversal or recirculation^46^.

**Fig. 2:**
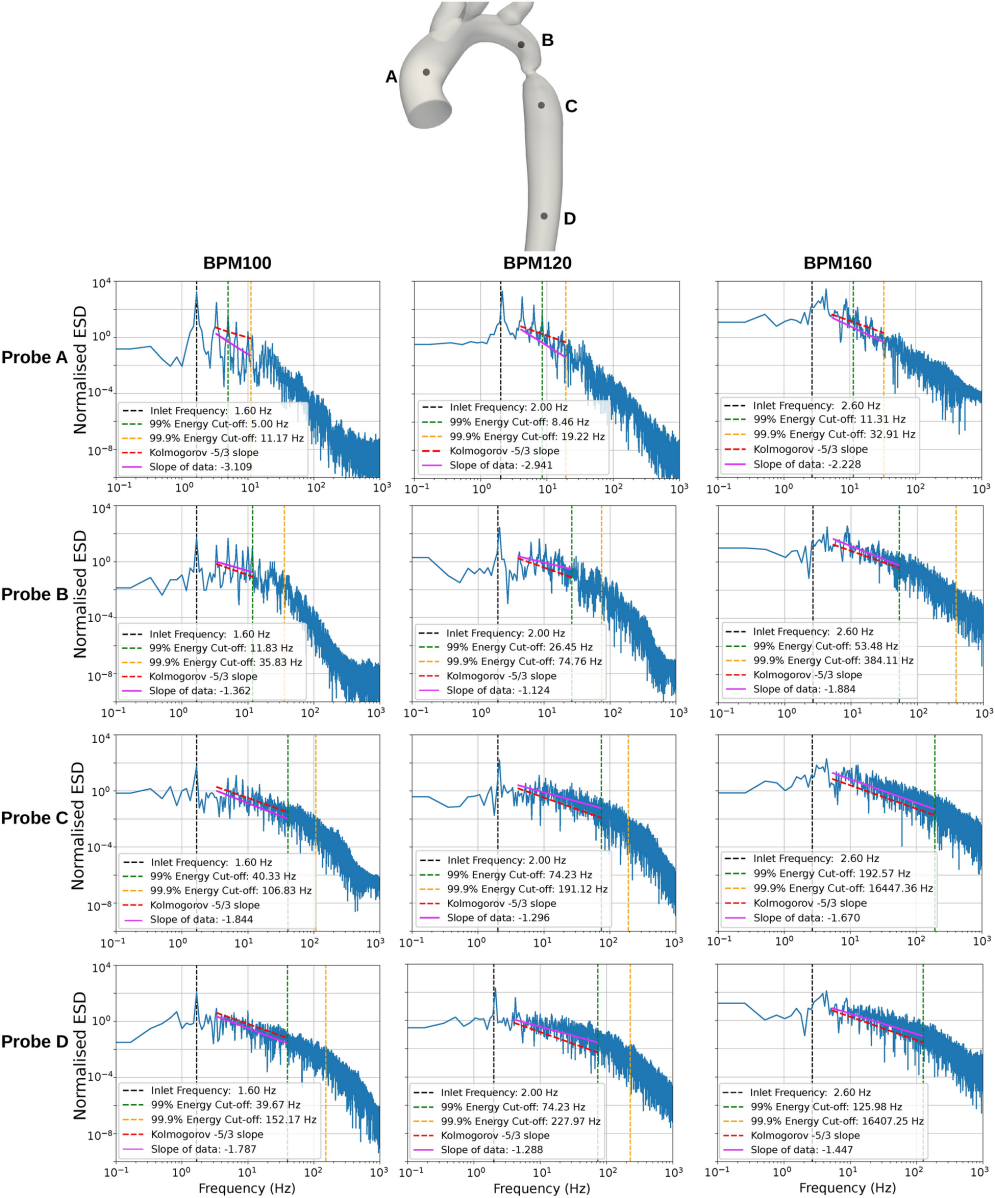
The normalised kinetic energy spectral density (ESD) at probe locations A, B, and C for BPM100, 120, and 160. A red dashed line indicates a $-5$/3 slope, while a green dashed line shows the cutoff frequency for 99% local energy. The orange dashed line marks spectra above 99.9%, helping to minimize focus on high-frequency noise. The yellow dashed line represents the inlet frequency for each BPM case.

To evaluate the statistical properties of turbulence in the aortic arch, velocity data were sampled over the final 20 cardiac cycles of the simulation at 4 different locations; (A) the ascending aorta (expected laminar flow), (B) upstream of the coarctation (transition to turbulence expected), (C) downstream of the coarctation (expected turbulent flow) and (D) in the descending thoracic aorta (moderate flow disturbances expected). The energy spectral density is calculated based on Eq. 6 and plotted in Fig. 2 to examine how KE is distributed over frequency scales. We calculated the slope of the linear cascade region in the energy spectrum and compared it to the theoretical values predicted by Kolmogorov’s scaling laws. In the plot, the frequencies corresponding to 99% and 99.9% of the total energy are also highlighted (Fig. 2).

As expected, turbulence increases from probe A to D along the aorta. At probe A, energy is observed to be predominantly concentrated at the frequency corresponding to the HR with minimal energy at higher frequencies. Moving to probe B, upstream of the coarctation, a slight increase is observed at higher frequencies suggesting the presence of flow disturbances and an increased flow unsteadiness. In contrast, the spectra at probes C and D, downstream of the CoA reveal a significant increase in energy across a broader range of higher frequencies, indicating developed turbulence. Specifically, mid-range frequencies show a prolonged alignment with the expected − 5/3 power law^47^, suggesting a longer interial cascade of turbulence, in line with higher local values of Reynolds number within the jet emanating from the coarctation. For the two lower HR, the dominant frequencies align well with their respective heart rates (1.67 Hz to 100BPM and 2 Hz to 120BPM), and are followed by consistent harmonic frequencies. This pattern reflects the periodic nature of the pulsatile flow. In contrast, at BPM160, the peak energy shifts towards a harmonic frequency, and displays a less-pronounced peak value, which could be due to the more complex nature of the turbulent flow dynamics at this HR. The higher peak systolic velocity at this HR results in a longer duration of the ‘jetting’ through the coarctation, which at this Reynolds number is less stable. Thus in this case the jet is more prone to fluctuations, resulting in a less coherent velocity profile, which is also observed in Fig. 1e for the same HR.

**Fig. 3:**
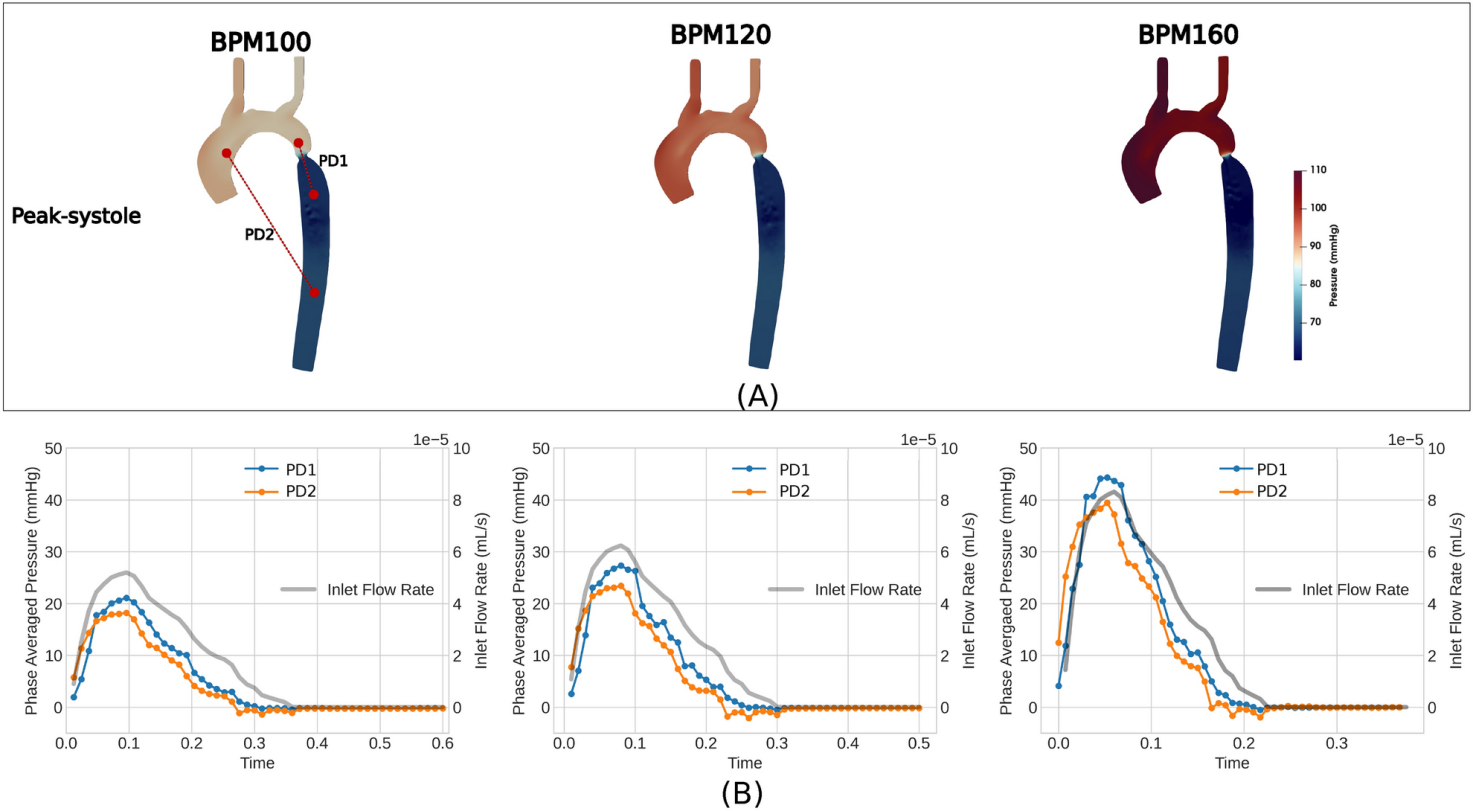
Pressure distribution during peak systole was examined at three HRs (**A**), showing two points for pressure drop measurement: PD1 near the coarctation site and PD2 between the ascending and descending aorta in the distal region. (**B**) Variations in PD1 and PD2 throughout the cardiac cycle for each BPM, with inlet flow rates plotted in gray for comparison.

In CoA, the difference in pressure at the site of coarctation is a clinically relevant hemodynamic parameter that provides a measure of the severity of the condition. Figure 3A shows the contours of the pressure at the peak of systole, and the pressure drop (PD) fields are referenced with a minimum value of zero. In the diagram, two pressure drop measurements are illustrated: PD1 represents the pressure variation between the locations just before and after the coarctation site, while PD2 indicates the pressure difference between the ascending and descending aorta (distal from the coarctation). The maximum pressure drops (PD1) throughout the coarctation site, namely 21.10, 27.32 and 42.28 (mmHg), for each HR, respectively, clearly exceed the standard threshold of 20 (mmHg) that indicates the need for intervention^5,48^. Given the clinical focus on this quantity, it is instructive to note how much this varies throughout the cycle and depending on where the measurement is taken. The evolution of PD1 and PD2 shows different patterns during cardiac cycles, and both reach their maximum drop when the inlet flow reaches its peak, as shown in Fig. 3B.The undulating variation of these quantities post peak systole is correlated to the dynamic motion of the jet, providing an example of uncertainty in extracting these measurements.

### KE vs TKE

**Fig. 4:**
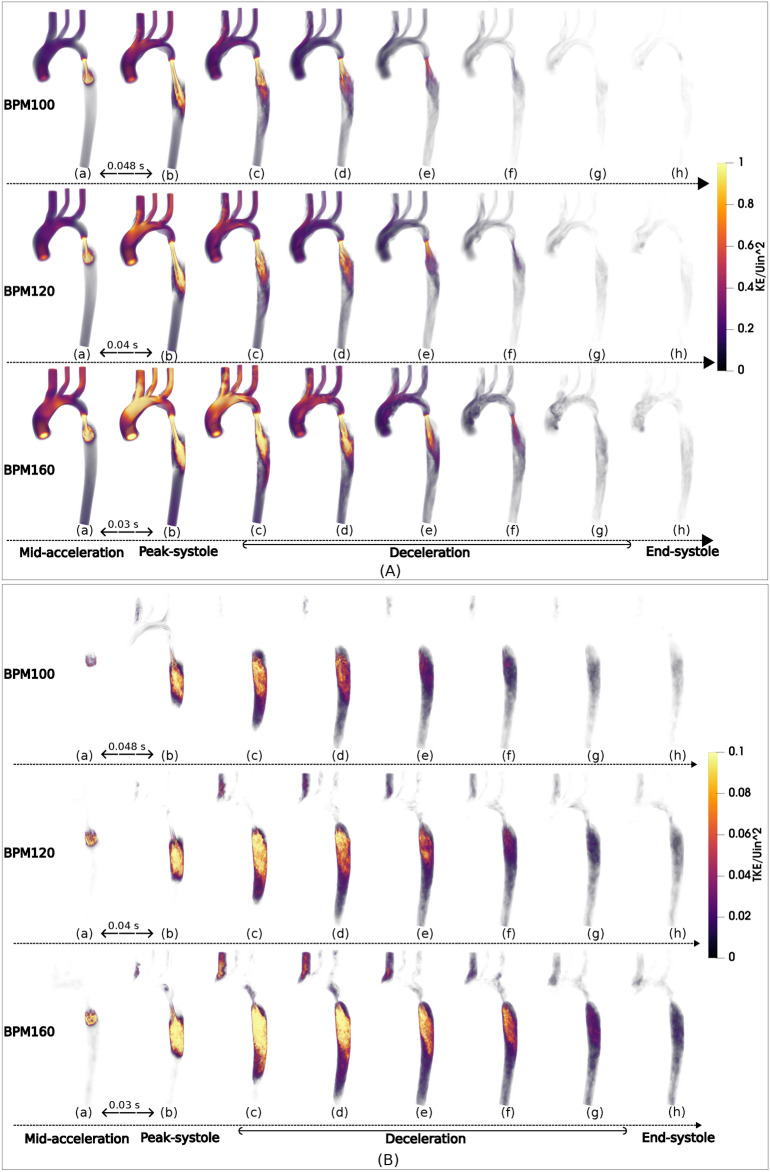
Volume rendering of normalised KE (**A**) and TKE (**B**) at different cardiac phases is shown for three HRs. Values are colour-coded normalised by inlet velocity of BPM120.

Kinetic energy (KE) can be decomposed into phase-averaged mean KE and phase-averaged turbulent kinetic energy (TKE) using Eqs. 10, 11. KE identifies high-energy regions that are associated with large-scale dominant-flow features, whereas TKE quantifies turbulent energy and is related to eddies in disturbed flows. Volume renderings of KE (Fig. 4A) and TKE (Fig. 4B) are plotted for each HR to illustrate flow and its turbulent behavior. KE and TKE are sampled at four time intervals over 50 intervals in a cardiac cycle: every 0.048 s for BPM100, every 0.04 s for BPM120, and every 0.03 s for BPM160. Note that peak systole is captured in the second sample plot, occurring at 0.096 s for BPM100, 0.08 s for BPM120, and 0.06 s for BPM160, respectively. Both values are normalised by the square of the maximum inlet velocity of the BPM120 case. The evolution of both KE and TKE demonstrate characteristic pattern of acceleration, peaking, followed by a period of steady deceleration gradually reaching zero values. The normalised KE is observed to reach a peak value that is approximately one order of magnitude larger than the normalised TKE. The TKE showed more spikes in acceleration (larger gradient) before reaching the peak value compared to the KE. Subsequently, the TKE decreased rapidly during the deceleration phase. The evolution of TKE lags slightly to that of KE, since the latter is generated as a consequence of the former having increased throughout the cycle. This observation is consistent with a previous comparison of MRI and LES by^15^. Therefore, to accurately assess the impact of turbulence throughout the cardiac cycles in CoA, it is important to consider not only the peak systole of the inlet but also the deceleration phase, where substantial turbulence is present in the descending aorta.

**Fig. 5:**
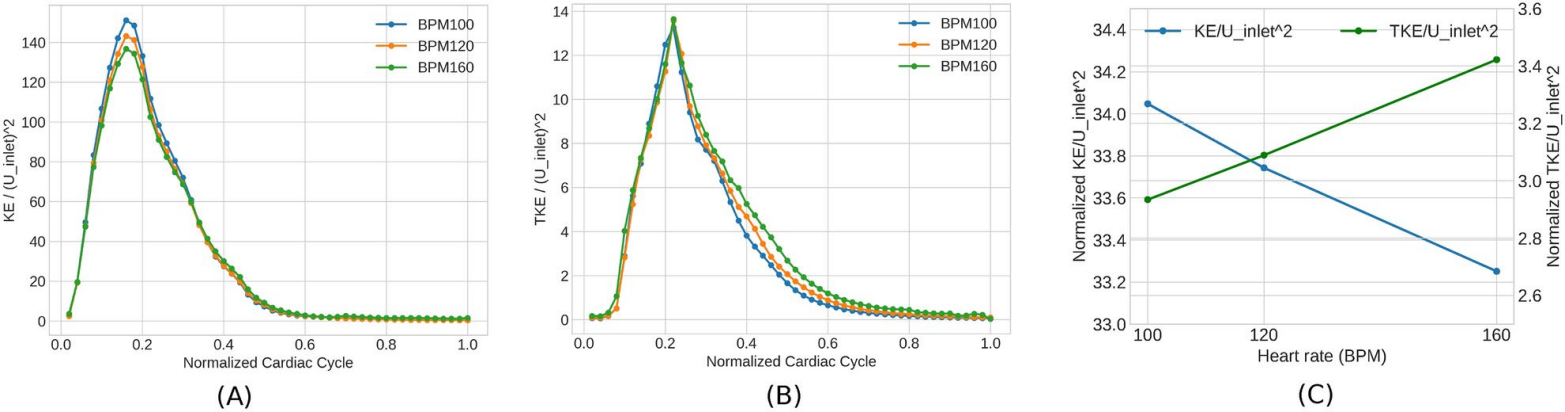
KE (**A**) and TKE (**B**) spatially averaged across the entire aorta are displayed and normalised for three HR scenarios. (**C**) The variations in maximum normalized KE and TKE across the three HRs are shown. Note that all figure values are normalised by the peak inlet velocity for BPM100, 120, and 160 respectively.

Figure 5A and B shows time-dependent integral values of KE and TKE, normalised by the squared inlet velocity of each BPM during a cardiac cycle. Note that the normalisation method used here differs from the volume rendering in Fig. 4, which offers a closer examination of the impact of HR on energy levels. It is instructive to note that the sum of the integral values of normalised KE and TKE are approximately constant for each HR; i.e. the sum of the area under the two graphs is conserved. For reference these values are, 36.7, 36.4 and 36.6 for 100 BPM, 120 BPM and 160 BPM, respectively. This can be interpreted that the transfer of energy from mean flow to turbulence is preserved. When HR increases from 100 BPM to 160 BPM, the normalised KE decreases at peak systole, while the normalised TKE increases, but only in the decelerating phase of systole. In all three cases, the KE peaks early, in line with a rapid increase in mean flow at the start of each heartbeat. On the other hand, the increase of turbulence in the deceleration phase is due to the greater rate of production of turbulence downstream of the CoA at higher HR. One might expected even normalised TKE to be higher at 160BPM compared to the other two HRs, particularly at the peak systolic value, however, this is not the case. As seen in the figure, overall normalised TKE displays similar values for all three HRs. This observation implies that the turbulence levels are predominantly influenced by the geometric features of the coarctation, rather than by changes in flow velocity associated with different HRs. These observations imply that flow energy efficiency decreases at higher HRs, i.e. the ratio of turbulence kinetic energy to total kinetic energy is greater. Figure 5C, presents a comparison of the energy variations in terms of the sum of the volume-integrated values of normalised KE and TKE, to understand the impact of varying HR of flow energy. With an increase in HR by 60%, the sum of normalised KE decreased by 2.3%, while the TKE increased by 16.6% from BPM100 to BPM160, with the sum of the variations is remaining constant.

### Wall shear stress patterns and its indicator

To account for the significant variability of WSS in both time and space, we provide three levels of WSS indicators: cycle-to-cycle averaged, spatially averaged, and phase-averaged WSS.

**Fig. 6:**
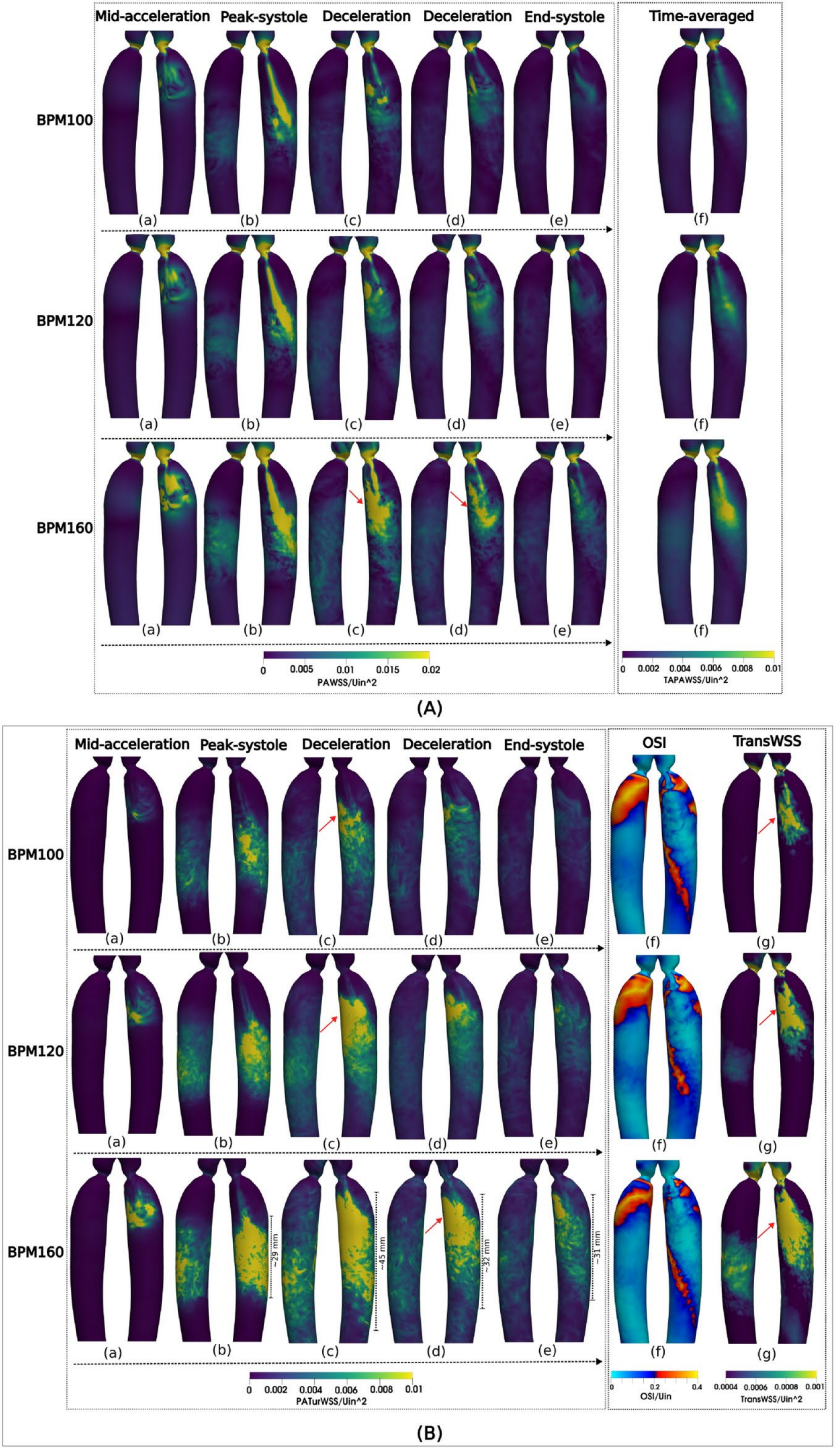
PAWSS (**A**) and TurWSS (**B**) values during different cardiac cycle phases (mid-acceleration, peak systole, deceleration, end-systole) for three HRs. The last column shows normalized TAWSS (**A**), TransWSS, and OSI (**B**). Values are color-coded normalised by BPM120’s max inlet velocity. Arrows point to highlighted text regions.

- *Time-averaged parameters* Typically presented and analyzed in studies on WSS, this approach provides a single value of the WSS experienced throughout the entire cardiac cycle and represents the average over multiple cardiac cycles. It includes parameters such as the cycle-to-cycle phase-averaged wall shear stress (TAWSS), the transverse WSS (TransWSS), and the oscillatory shear index (OSI). The equations used to compute these quantities are normalised by the inlet velocity of BPM120, as shown in Fig. 6A:(f) and B(f) and (g).
- *Phase-averaged parameters* PAWSS and its turbulent component turbulent WSS (TurWSS), are calculated using Eqs. 13 and 14. Phase-averaged parameters represent the average at a specific time within the cardiac cycle, e.g., acceleration, peak systole, deceleration, and end systole. The values are normalised by the input velocity of the baseline BPM120 and are shown in Fig. 6A:(a)–(e) and B:(a)–(e).
- *Spatially averaged analysis* This approach applies spatially averaged analysis by of PAWSS and TurWSS dividing the CoA into two segments to illustrate spatial differences. The results are displayed in a 3D anatomy with 2D front and back views, providing a comprehensive visualisation of the spatial variations in WSS.

The absolute maximum and mean values of the wall shear stress indicators are summarised in Tables 1 and 2. WSS-based indicators show both variations and similarities when compared. The comparative analysis of the WSS indicators reveals key insights into the hemodynamic environment prevalent in the vascular regions studied.

**Table 1:** Mean value WSS’s indicator over cardiac cycles.

	BPM100	BPM120	BPM160
TAWSS (Pa)	8.24	9.88	14.15
TranWSS ($\times 10^{-4}$ Pa)	7.61	9.90	15.47
OSI (-)	0.18	0.17	0.15

**Table 2:** Maximum value of WSS’s indicator over cardiac cycles.

	BPM100	BPM120	BPM160
Peak PAWSS (Pa)	205	246.08	347.55
Peak TurWSS (Pa)	44.92	64.58	59.92
TAWSS (Pa)	56.8	70.6	104
TranWSS (Pa)	3.56	4.61	6.76
OSI (-)	0.5	0.5	0.5

#### PAWSS and its temporal-averaged indicators

PAWSS, TurWSS, TAWSS, TransWSS, and OSI are shown in Fig. 6. All three HR simulations show similar temporal patterns of magnitude of PAWSS. During acceleration, elevated PAWSS is observed on the inner side of the descending aorta, a result of the development of jet flow in the initial stage. At peak systole, a jet-shaped high PAWSS form predominates, affecting mainly one side of the arterial wall, while the opposite side displays lower PAWSS values. The decrease in inlet flow diffuses the PAWSS value across a larger region of the descending aorta as the flow jet subsides. During the diastolic phase, the magnitude of PAWSS is significantly reduced, but becomes more spatially divergent, especially in the TurWSS contour, as shown in Fig. 6A(a)–(e). The magnitude of PAWSS increases with increased HR, which is quite similar to the character of the jet flow captured by KE. TAWSS are presented alongside PAWSS to facilitate comparison. In particular, normalised TAWSS values are elevated in specific regions of the arterial wall, which is consistent with the characteristics of jet flow but have lower values than PAWSS. As HR increases, the impact of TAWSS extends to larger areas. However, because of the numerical averaging method employed, certain intricate details of WSS are not captured as it smooths out the instantaneous fluctuations.

In comparison, high TurWSS is distributed downstream and is less spatially concentrated, as shown in Fig. 6B. Unlike the jet-like pattern of PAWSS, TurWSS has a greater impact on the descending aorta, extending closer to the thoracic aorta, distal to the descending aorta. TurWSS spreads even further across the entire descending aorta during deceleration. Furthermore, an increase in HR results in a higher impact of WSS, elevated turbulence also increased both in magnitude and in spatial variability. Regions with elevated TurWSS exhibit larger and more circumferential effects. The approximate extent of TurWSS is quantified in Fig. 6B at BPM160. TurWSS is observed to propagate from the stenosis to the distal segment of the descending aorta with a jet in Fig. 6B:(a)–(b). Subsequently, it initiates slightly beyond the distal part of the descending aorta and extends retrograde toward the stenosis, diffusing at the proximal aorta.

OSI and normalised TransWSS provide both the nature and characteristics of disturbed flow. It should be noted that OSI is already a non-dimensional quantity according to its definition. The disparities between HRs arise mainly from the varying magnitudes of the indicators, as presented in Tables 1 and 2, while some similarities are observed in the spatial patterns. An OSI value of 0.2 can be considered a medium value that serves as a threshold separating regions of low and high OSI. Regions with OSI values below 0.2 are typically associated with relatively stable and unidirectional blood flow, indicating lower levels of flow disturbance. In contrast, OSI values above 0.2, particularly those approaching 0.5, suggest a significant oscillation in the WSS. High OSI values are often accompanied by low TAWSS and TurWSS values. These high OSI values are the result of specific flow patterns that exhibit alternating positive and negative WSS values, which cannot be calculated with a time-averaged indicator such as TAWSS, eventually cancel out each other over time. Normalised and absolute OSI patterns show continuous blade-shaped forms that traverse both the front and the back of the aortic wall. This is the region where the impact of the jet hit and the jet dissipated Fig. 6B:(f). Furthermore, regions of high normalised OSI decrease as HR increases. This decrease indicates a reduction in bidirectional flow and a reversal of the flow during the cardiac cycle. However, the stronger oscillation observed in the high HR scenario is not reflected in the OSI. Compared to instantaneous values, the time-averaged parameters exhibit significant differences in both time and space, following similar patterns.

On the contrary, transWSS reveals transverse shear stress, which can be indicative of swirling or vortex-like flow patterns. Figure 6B:(g) illustrates a contrasting pattern in which high TurWSS is accompanied by low OSI. This observation suggests that TranWSS is noticeable in the regions situated between the proximal ascending WSS (PAWSS) and TurWSS, demonstrating a closer similarity to disrupted TurWSS during the deceleration phase and displaying a more circular configuration. Across different HRs, there are consistent transWSS patterns, with a specific instance of TurWSS highlighted with arrows, indicating that transWSS reflect the disruptive effects of both average and fluctuating WSS. However, the impact of TurWSS on the distal descending aorta is not clearly evident within the transWSS. The magnitude of TurWSS increases with higher HRs, yet similar patterns persist.

#### Spatial-averaged WSS

**Fig. 7:**
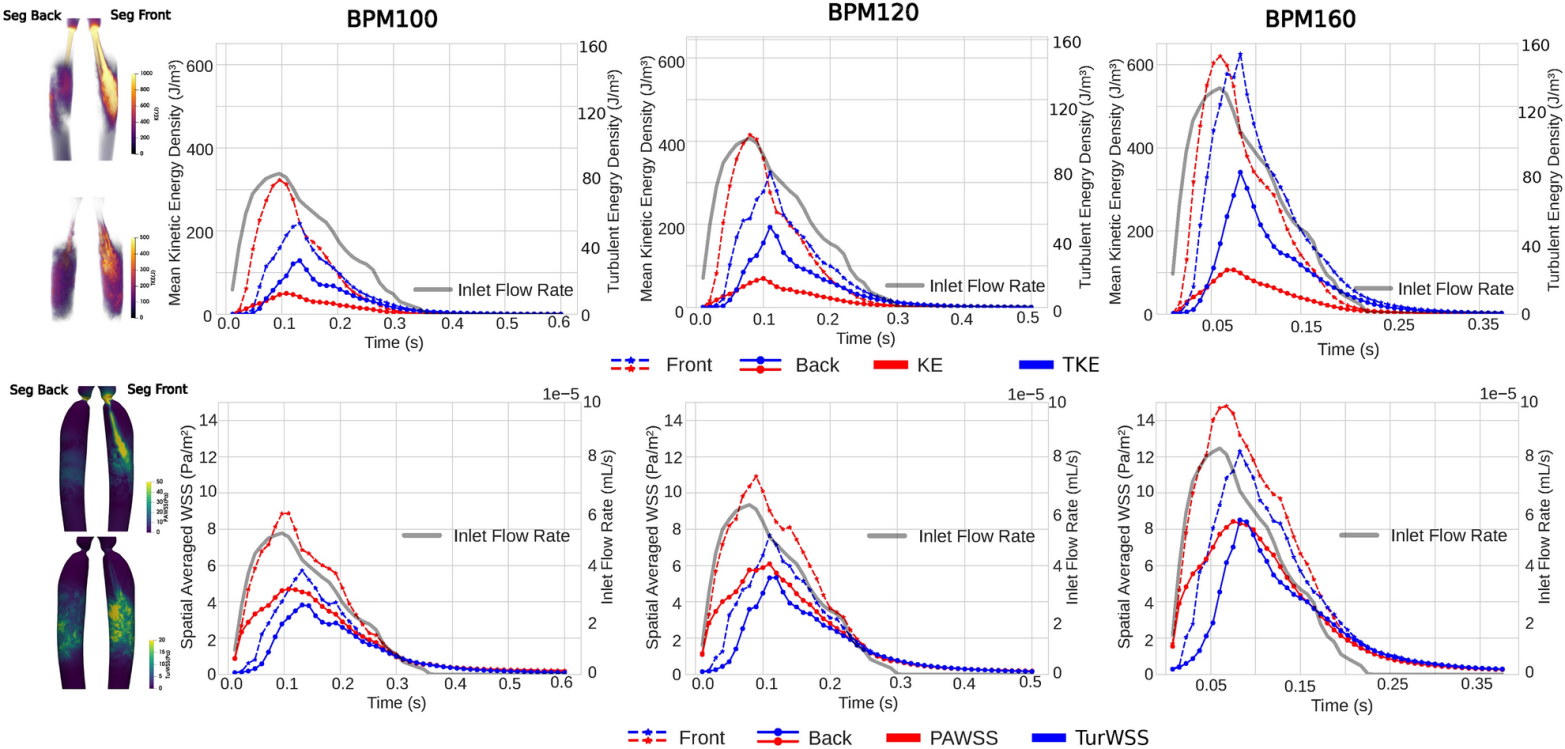
The CoA was split into two sections: Seg Back and Seg Front, based on the jet flow direction shown on the left in Figures. (Top) Average spatial KE and TKE of the divided regions throughout the cardiac cycle at various HRs. (Bottom) Average spatial PAWSS and TurWSS of the divided surfaces throughout the cardiac cycle at different HRs.Note: It should be noted that the outcomes presented may vary depending on the segmentation approach and presentation method.The goal is to highlight the presence of asymmetric jet flow and its potential occurrence in various patient-specific geometries. The front and back regions are divided with a volume ratio of approximately 5:4, while having the same surface area.

The area of interest, the coarctation site, was divided into two parts: Seg-Front, which was the most affected by jet flow, and Seg-Back, which covered the remaining area (Fig. 7). In order to illustrate the eccentricity of the flow and the asymmetry of the WSS in this scenario, an arbitrary segmentation method was employed based on the author’s observation of the jet flow. However, efforts were made to maintain an equal surface area between the front and back (approximately 8 cm^2^). The energy is computed based on the volumes of two segments and the WSS of the surface as the mean spatial absolute values. The KE and TKE per volume unit, PAWSS and TurWSS per surface unit are determined throughout the cardiac cycle as shown in Fig. 7. This enabled the assessment of the impact of PAWSS and TurWSS on particular areas over a period of time, as well as their connection to energy.

The spatially averaged TAWSS in Seg-Front consistently exceeded that of Seg-Back in all three cases, and Seg-Front experiences approximately twice as much Seg-Back. In most cases, PAWSS is the main contributor of WSS compared to TurWSS. However, as expected in BPM160, TurWSS slightly exceeds PAWSS. The difference between TurWSS and PAWSS decreases as HR increases. As HRs increase, Seg-Back encounters rapid growth in TurWSS compared to PAWSS, and both reach the same maximum value at BPM160. This is illustrated in the evolution of TurWSS shown in Fig. 6B, where high TurWSS leads to expansion of the descending aorta during the deceleration phase. This disrupts the flow dominated by Seg-Back and reduces the impact of the jet on PAWSS. In contrast, there is a notable disparity in energy density between the front and the back, with the energy at the front being ten times greater than that at the back. Clearly, as a result of the impact of the jet, most of the KE and TKE asymmetrically affect one side of the geometry. As a result, this leads to an uneven distribution of WSS. The variances in TKE between the front and back regions increase with increasing HR, a trend similar to that observed in the WSS pattern. This trend could be attributed to the higher Reynolds number resulting from the elevated inlet boundary condition, which leads to a more pronounced turbulence. Seg-Front was observed to dominate in both types of WSS as HR varied. Furthermore, the distinct spatial patterns of the two types of WSS exhibited contrasting distribution trends with changes in HR. To show the difference in localized WSS experience in time, the absolute PAWSS and TurWSS time and spatial means for the Seg front and the Seg back were summarized in Table 3.

**Table 3:** Summary of the time and spatial averaged of Seg Front and Seg Back surface for PAWSS and TurWSS in each cases.

(Pa m^2^) per cycle	BPM100	BPM120	BPM160
Front PAWSS	2.63	3.30	5.07
Back PAWSS	1.69	2.12	3.27
Front TurWSS	1.55	2.14	3.66
Back TurWSS	1.13	1.53	2.44

The cyclic nature of the flow is readily apparent, as is the anticipated phase shift between the mean KE and the maximum TKE. As the flow passes through the coarctation and enters the post-coarctation region, the initially laminar or weakly turbulent flow transitions into a more intense turbulent state, leading to this observable delay. Turbulence increases with time, especially during the later stages of the cardiac cycle, and is mainly observed in the descending aorta, downstream of the coarctation. These findings are further corroborated by a recent experimental investigation of stenotic carotid models^13,49^. When HR rises, turbulence development does not develop directly at the peak of systole, but rather occurs over time, particularly during the deceleration phase, highlighting that TKE does not increase by the same extent during peak systole as it does during deceleration. Meanwhile, inconsistency in time and space between KE and TKE influences the related results of PAWSS and TurWSS, which presents the strong positive correlation between KE&TKE and PAWSS&TurWSS.

**Table 4:** Key parameters of interest for each BPM.

BPM	Mean Re number	Max Re number	Max pressure Drop	Max velocity	Max Integrated KE	Max Integrated TKE	TKE / (TKE+KE)	TurWSS/total WSS
	–	–	(mmHg)	(m/s)	(Pa)	(Pa)	(–)	(–)
100	632	2138	21.10	2.37	169.86	14.89	7.4%	33.2%
120	902	2462	27.32	2.73	234.62	22.22	8.9%	35.2%
160	1172	3103	49.28	3.44	395.31	39.42	9.5%	36%

The mean & max Reynolds number (Calculated based on the radius at the coarctation site and the mean (cycle-averaged) and maximum (peak systolic) velocities at that site), maximum pressure drop and velocity occur in the coarctation region. The max integrated KE and TKE over the aorta. The ratios of TKE to total energy (KE+TKE) and turbulent WSS to total WSS are integrated throughout the aortic domain. Note that the ratios are calculated on the basis of absolute values.

Table 4 summarises the relationship between HR and key hemodynamic metrics. With increasing HR, the maximum velocity increases from 2.37 ms^−1^ to 3.44 ms^−1^. Higher HR accelerates blood flow through coarctation, leading to a notable increase in velocity and pressure drop, resulting in higher resistive forces encountered by blood flow. Clearly, a higher HR leads to higher energy for mean and turbulent energy. However, when normalizing the total TKE by the input energy, we observe an inverse relationship between normalized TKE and HR. Although the absolute value of TKE increases with HR, the proportion of input energy converted into turbulence decreases as HR increases. This suggests that at lower HRs, a higher proportion of the input energy is dissipated as turbulence, indicating decreased energy efficiency. A similar observation of a less significant reduction in TKE with increased dilation was made by Andersson et al.^19^. Furthermore, as shown in Table 4 the ratio of TKE to total KE increases slightly with HR, going from 7.4$\%$ at 100 BPM to 9.5$\%$ at 160 BPM. Similarly, the ratio of the absolute shear stress of the turbulence wall to the total absolute shear stress of the wall also increases modestly from 33.2$\%$ at 100 BPM to 36.0$\%$ at 160 BPM. An increase in the relative level of turbulence present is expected at higher HR, but the increase is not proportionate. From Table 4, a 45% increase in max velocity (from 2.37 to 3.44) corresponds to an increase in TKE ratio of 28% and an increase in TurWSS ratio of just 8%. While TurWSS remains a significant contributor to total wall shear stress in the coarctation zone, justifying the need for a scale resolving approach, in this case it is the stenosis itself which drives the bulk of the turbulence, and HR reduction does not proportionally reduce turbulence in the disturbed flow regions. It should also be noted that near wall levels of resolved turbulence are highly sensitive to mesh resolution in that region and as such this value may be higher than observed here. While in the present work 95% of turbulence is resolved in the region downstream of the coarctation (see supplementary material), this may not be providing a clear picture of the levels of near wall resolution, since even where all scales are resolved, their net contribution may be a low fraction of the overall KE in the flow.

## Discussion

This research explores how blood flow demonstrates turbulent behavior, especially during transitional flow, a notion supported by theoretical validation and in vivo^30,50,51^. In particular the purpose is to investigate the effect of different HRs on CoA, in line with the observations and motivations of Andersson et al. and Riva et al.^52,53^. Given the focus on turbulence, a scale resolving approach such as LES is necessary in order to capture the broadband dynamics, particularly in the decending aorta^29^. Here, high-fidelity simulations are performed, for sufficient duration to obtain phase-average results to explore the impact of HR on aortic flow and the potential effects of pharmacological interventions on hemodynamic parameters, which act to modify HR.

In coarctation, the flow is accelerated through a stenosed region of the aorta, which particularly during systole leads to an increase in velocity and a subsequent ’transition’ from laminar to turbulent states. The relationship between HR and key hemodynamic metrics is explored, showing how higher HRs intensify blood flow through coarctation. Specifically, the Reynolds number at the coarctation site varies with HR as shown in Table 4. Previous work reported significant heritability of factors leading to high mean Reynolds numbers in arterial flow^54^. This correlation suggests that individual genetic differences could affect the severity of turbulent flow in coarctation patients, potentially impacting clinical outcomes.

In the patient-specific geometry tested, we observed that the jet switched cyclically between peak systole and the deceleration phase, shifting from impingement on the front (inner) side to the back (outer) side of the descending aorta. The disturbed flow region at the post-coarctation site formed as the jet developed and dissipated (supplementary video provided). Spectral analysis indicated that lower HRs do not significantlt impact the turbulence formation through the stenosis, though turbulence upstream is affected as seen in Fig. 2—Probe A. At higher HRs (160BPM), turbulence initiates upstream of the CoA near the aortic arch branches due to sharp directional changes and acceleration, forming helical flow patterns consistent with previous findings^13,55,56^. While higher HR influences recirculation zones, Fig. 1, their locations are predominantly determined by the coarctation geometry. Turbulence increases with HR but less so than variation in velocity and pressure drop.

Disturbed blood flow can cause endothelial trauma, increase the risk of aneurysm formation, thrombosis, and vascular remodeling^57–59^. Our study emphasized the HR variability’s influence on PAWSS maximum and minimum as seen in Tables 1 and 2. Therapies reducing HR are expected to alleviate related hemodynamic stresses. Rvia et al. demonstrated that dobutamine-induced stress increased TKE in the aorta, suggesting greater level of turbulence and less efficient blood flow^53^. We observed that the ratio of total TKE to total KE reduced with HR, indicating increased energetic efficiency despite reduced turbulence.

Turbulent flow conditions result in a broader distribution of WSS directionality, prompting a reevaluation of WSS metrics to effectively capture the unsteady, multidirectional stress field. PAWSS and TurWSS are time-varying metrics providing insights into WSS magnitudes at critical moments like peak systole. TAWSS offers a long-term view but lacks the detail of PAWSS. TransWSS evaluates shear stress perpendicular to the main flow, relevant in areas with secondary flows, while the oscillatory shear index OSI quantifies directional changes in WSS, linking high OSI values to flow reversal and atherosclerosis^60^.

Comparing these metrics (Fig. 6) highlights the importance of selecting appropriate WSS analyses in CoA. TurWSS showed insensitivity to HR reductions; even with significant HR decreases, turbulence-induced shear stress remains. This suggests that therapies solely reducing HR may not fully mitigate turbulent shear stress in CoA patients. TransWSS effectively identifies areas of high PAWSS but places less emphasis on maxima and minima due to its basis on an averaged flow field. Regions where turbulence occurs briefly during the cardiac cycle, such as the lower descending aorta, may not display high TransWSS values. Instead, elevated TransWSS is found between regions of peak time-averaged WSS and fluctuating TurWSS because it aggregates directional WSS throughout the cardiac cycle, capturing multidirectional vectors from both high mean WSS and turbulence-induced fluctuations^61^. However, the accuracy of TransWSS decreases in areas with complex or transient flow behavior, as it may not fully capture PAWSS, especially where turbulence peaks briefly. This limitation is significant in regions with rapidly changing flow directions or saddle points in the WSS vector field, where TransWSS may produce misleading results, as noted by Andersson et al.^52^. Additionally, the distribution of high TransWSS changes with disturbed flow; flow disturbances caused by different HRs interact non-linearly with existing patterns, altering the local shear stress environment. Future work could explore using the phase-averaged velocity field to compute TransWSS.

The OSI is effective in identifying regions at risk for vascular disease, often corresponding to turbulence areas when plotted for values below 0.25. While high OSI values are associated with disturbed flow and disease development, they do not always correlate with high turbulence levels; elevated OSI can occur in regions with significant flow reversal and low TurWSS. Studies have reported streaks of low WSS and high OSI at various anatomical locations, which are associated with areas prone to disease development, as seen in animal models studying atherosclerosis^61^. Traditionally, high OSI values have been indicators of disturbed flow regions linked to atherosclerosis and endothelial dysfunction, especially at arterial bifurcations^62^. However, there is growing recognition that low OSI values can also correlate with disturbed flow in low-shear, unidirectional environments, potentially disrupting endothelial cell function over time^62,63^.

Integrating TAWSS, OSI, and TransWSS is crucial for describing flow conditions in CoA geometry influenced by high jet velocity or oscillatory flow. These metrics highlight variations in WSS, indicating areas prone to plaque formation, endothelial dysfunction, or surgical intervention. Each captures different WSS aspects: OSI focuses on oscillatory shear stress during the cardiac cycle, while TransWSS reveals multidirectional shear stress vectors and complex 3D flow patterns. Using both together provides a more comprehensive understanding. Future studies may benefit from combining TransWSS with OSI for complementary insights, especially in regions of high turbulence or multidirectional flow.

The observed insensitivity of TurWSS to HR reductions is also highlighted. At lower HR levels, the cardiac cycle’s deceleration phase lengthens, allowing more turbulence to develop. Higher BPM leads to a higher peak systolic flow rate, and greater levels of turbulence, but in the case of CoA it is the stenosis itself which drives the bulk of the turbulence, and HR reduction does not proportionally reduce turbulence in the disturbed flow regions. From Table 4, a 45% increase in max velocity (from 2.37 to 3.44) corresponds to an increase in TKE ratio of 28% and an increase in TurWSS ratio of just 8%. The latter measurement may be particularly sensitive to mesh resolution, but nevertheless this is an important finding. It demonstrates that therapeutic strategies aimed at reducing HR may not fully alleviate turbulence-induced shear stress, especially in patients with aortic coarctation or other vascular abnormalities.

These insights are valuable when considering therapeutic strategies aimed at mitigating adverse hemodynamic stresses. The choice of therapeutic agent is influenced by the age of the patient, the associated pathologies, and the preference of the physician. The significance of beta-blockers, not only in adult heart failure management but also for potential pediatric benefits, requires further investigation of their specific impacts on children with CoA^5,42^. To date, virtual medical treatments are becoming increasingly viable, as demonstrated by research on beta-blockers in type B aortic dissections performed by Abazari et al.^64^. Our findings indicate that substantial HR reductions do not proportionally decrease turbulence-induced forces on arterial walls, posing ongoing hemodynamic challenges despite intervention.

This research is particularly relevant to understand the dynamics at play in arteries and remodelled vessels under turbulent flow conditions, especially after stenosis, where the impacts of jet flow are significant^65^. Even with substantial HR reduction, turbulent forces exerted on arterial walls continue to pose a hemodynamic challenge. This has implications for therapeutic strategies that aim to reduce HR, as they may not fully alleviate turbulence-induced shear stress, particularly in patients with aortic coarctation or other vascular anomalies. Ultimately, a regional examination was performed to analyses the distribution of energy and WSS between the front and back regions. The differences observed between the front and back regions are substantial. This is because the consistent jet pattern and the characteristic of a notably irregular wall interaction are less prominent when the HR varies. Consequently, this could potentially result in subsequent remodeling processes that can further alter the dynamics of hemodynamic flow, regardless of HR^66,67^.

We made necessary simplifications. A significant one is assuming the aortic wall is rigid, which overestimates backflow near the wall. Modeling fluid-structure interaction with moving vessel walls is possible but costly and complex due to challenges in obtaining accurate pediatric biomechanical properties. Thus, vessel wall elasticity wasn’t considered, affecting flow dynamics accuracy, especially in compliant vessels. However, including elasticity wouldn’t significantly alter key insights into flow behavior, particularly away from the wall where wall motion influence is minimal^68^. A further limitation arises due to the lack of specific patient data, a fixed flow split rate at outlet, as used in the present work, may not fully represent physiological conditions. The use of patient-specific flow rates under varying HR would be preferable, where available. Related to this is the absence of 4D flow MRI data, which would have provided more detailed insight into boundary conditions and flow patterns, particularly the flow and the unsteadiness arising from the aortic valves, which is not included in this work. Finally, while in the present work the focus has been necessarily placed a single geometry, in order to afford careful comparison of methods, expansion of these assessments to include multiple patients would allow for more statistically significant conclusions to be drawn.

## Conclusion

The study investigates the impact of three different HRs on hemodynamics in a patient with CoA, a condition associated with an unorthodox flow field and high vessel wall load and potential complications from drug treatment. High-fidelity simulations were undertaken and careful comparisons were made of a range of pertinent flow metrics to assess differences. The focus on methodology in this study emphasises not only the potential of large eddy simulation, but also the need for careful evaluation of mesh resolution. As expected, elevated HR leads to higher levels of turbulence downstream of the stenosis, leading to an inverse relationship between flow efficiency and HR, suggesting that modulating HR could be a viable therapeutic strategy to alleviate hemodynamic loading on vessel walls.

In particular, higher HR was observed to lead to notably elevated levels of fluctuating wall shear stress (TurWSS) which is associated with endothelial damage and dysfunction. Higher HR was correlated to increase in all measures of WSS, TKE, and pressure drop. In particular TAWSS increasing from 8.24 Pa to 14.15 Pa and TKE from 14.89 Pa to 39.42 Pa between 100 BPM and 160 BPM. However, we also observed that the ratio of TurWSS to total WSS remained relatively stable across different HRs, indicating that the presence of the CoA itself is more of a drive for the turbulence in the descending aorta than the change in blood flow rate itself. Lowering of the HR alone may not significantly diminish the turbulent forces exerted on arterial walls. This highlights the need for comprehensive approaches that address both HR modulation and flow disturbances, i.e. via corrective surgery. While such high-fidelity simulation is rarely used in clinical decision making at present, this study serves to demonstrate the role that detailed examination of complex flow physics can play in helping to identify regions of elevated WSS and may help lead to improved understanding of the mechanisms of arterial disease.

## Materials and methods

### Patient recruitment and image acquisition

**Fig. 8:**
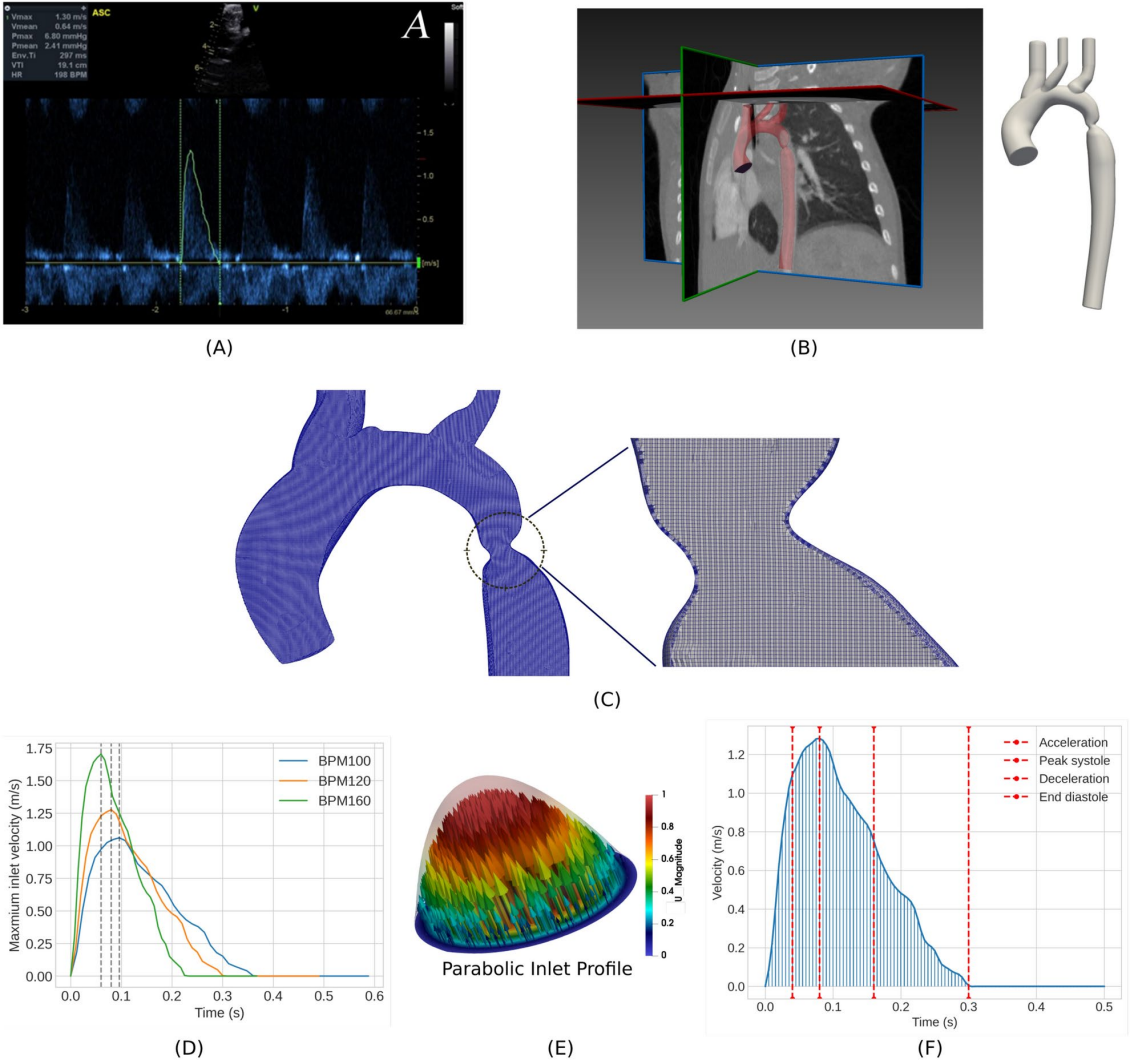
Processing patient data. (**A**) An illustrative Doppler echocardiography dataset captured at the ascending aorta with digitised velocity-time plot. (**B**) CTA images and segmented CoA geometry. (**C**) Mesh sample including close-up of the coarctation region. (**D**) Inlet flow rate waveform for 100, 120, and 160 BPM. (**E**) Example plot of the parabolic inlet profile of BPM120. (**F**) Inlet peak velocity demo of BPM120 with 50 time bins and key time phase.

An 18-month-old infant with aortic coarctation was scanned at the Red Cross War Memorial Children’s Hospital (RXH), Cape Town. A data collection protocol was established for CoA patients in low- middle-income countries based on access to infrastructure and standard clinical protocols. In such countries, access to advanced imaging modalities, such as magnetic resonance imaging, is limited or unavailable. The established imaging protocol is based on computed tomography angiography (CTA) for geometric quantification and Doppler echocardiography for velocity-based quantification. Details of the complete clinical protocol are described in our previous study^69^. CTA was acquired with a scan time of 714 mm, slice thickness of 0.5 mm and uniform pixel spacing of 0.3691 mm. The aortic geometry was segmented from CTA using open-source software SimVascular. The aorta was segmented from the level of the sinotubular junction in the ascending aorta to the descending thoracic aorta and included the innominate, the left common carotid, and the left subclavian arteries, as shown in Fig. 8B. Pulsed-wave Doppler echocardiography measurements were acquired at the site of the cardiac valve junction, which corresponds to the inlet of the computational model. Doppler echo provides velocity measurements at a fixed spatial location over multiple cardiac cycles. This study is in accordance with the approved ethical guidelines, and the data used in the present and previous study^69^ were reviewed and approved by the Ethics Committee of the University of Cape Town (HREC Reference R017/2014).

### Computational mesh details

An unstructured hexahedral mesh was generated using the open-source tool; snappyHexMesh. A mesh sensitivity test was performed using the highest HR that corresponds to the highest Reynolds number. The results are presented in the supplementary material. After careful consideration, an unstructured hexahedral mesh consisting of 8.2 million cells was chosen, as it exhibited a well-convergent velocity field compared to a mesh with 10 million cells. Figure 8C shows the mesh examples used in this work.

### Boundary conditions

A pulsatile, parabolic velocity profile was imposed at the inlet, according to the Doppler echocardiography measurement, as shown in Fig. 8A. Raw velocity data are processed to enable compatibility with CFD simulation, including smoothing and normalisation (Plot Digitiser software) of the HR to 120 BPM. Full details of Doppler echocardiography data processing can be found in^69^. The cross-sectional area of the aorta is determined from the segmented geometry and is assumed to remain constant throughout the cardiac cycle. At the aortic inlet, the area is 99.63 mm^2^. Processed Doppler echocardiography at 120 BPM is multiplied by the cross-sectional area to give a time-varying flow rate representative of the patient’s cardiac cycle (Fig. 8D). In the computational model, this is then prescribed as a parabolic profile inlet boundary condition as shown in Fig. 8E.

The resting HR range for a 1-year-old child is typically between 80 and 130 beats per minute^70^. However, in the case of CoA, the HR may be higher due to the increased workload of the heart^71^. To evaluate the effects of HR on CoA hemodynamics, we consider three different HR of 100, 120 and 160 BPM which represent medically lower, at rest, and elevated states, respectively. In the absence of Doppler echocardiography measurements for higher and lower HRs, we modified the baseline flow rate of 120 BPM by assuming a constant stroke volume, where the amount of blood ejected from the left ventricle remained fixed over one cardiac cycle^72^. Therefore, HR and cardiac output are inversely proportional in this study. The flow rate waveforms for each BPM are shown in Fig. 8D and the mean/maximum velocity over a cycle for 120 BPM is shown in Fig. 8F, highlighting key times in the cardiac cycle.

The three-element Windkessel model (3EWK) was applied to the descending aorta and the three outlets of the branches. Windkessel parameters were calculated using Doppler-measured flow rates for each outlet as detailed in^69^. Note that in the healthy aorta, approximately 30$\%$ of flow leaves the three branches of the arch; however, in patients with CoA, this value can increase drastically. In the present case, 72.3$\%$ of the flow is directed toward the arch branches. Obtaining the mean arterial pressure is necessary for patient-specific model calibration. Since pressure readings were not obtained for this patient, a value of 123/60 mmHg was assumed based on published data on pediatric CoA^73^. The coefficients of proximal resistance, distal resistance, and arterial compliance used in the 3EWK model can be found in the Supplementary Material. The walls were assumed to be rigid and nonslip, which is a common assumption in many CFD studies, particularly those focusing on hemodynamics and blood flow patterns^74^.

### Numerical model

This study employs LES for incompressible flow to model the transition from laminar to turbulent flow in a pulsatile environment. The wall-adapting local eddy viscosity (WALE) subgrid model is selected, with 99% near-wall y+ values below 1, and all below 5, to precisely capture flow dynamics near solid boundaries. The filtered continuity and momentum equations, along with the WALE strain-rate tensor model, allow for a detailed analysis of the turbulent structures. This modelling approach has been used in previous studies of cardiovascular flows^15,19,75^. Blood shows non-Newtonian characteristics in small capillaries, but larger vessels use a Newtonian fluid model instead^76^.The filtered continuity and momentum equations for an incompressible fluid are given by:

1 $$\frac{{\partial \bar{u}_{i} }}{{\partial x_{i} }} = 0$$

2 $$\begin{aligned} & \frac{\partial {\bar{u}}_i}{\partial t} + {\bar{u}}_j \frac{\partial {\bar{u}}_i}{\partial x_j} = -\frac{1}{\rho } \frac{\partial {\bar{p}}}{\partial x_i} + \nu \frac{\partial ^2 {\bar{u}}_i}{\partial x_j \partial x_j} - \frac{\partial \tau _{ij}^{sgs}}{\partial x_j} \end{aligned}$$

where, ${\bar{u}}_i$ represents the filtered velocity, ${\bar{p}}$ is the filtered pressure, $\rho$ is the fluid density, $\nu$ is the kinematic viscosity. $\tau _{ij}^{sgs}$ is the subgrid-scale stress tensor:

3 $$\begin{aligned} & \tau _{ij}^{sgs} = 2 \nu _{t} {\bar{S}}_{ij} + \frac{1}{3} \tau _{kk} \delta _{ij} \end{aligned}$$

4 $$\begin{aligned} & {\bar{S}}_{ij} = \frac{1}{2} \left( \frac{\partial {\bar{u}}_i}{\partial x_j} + \frac{\partial {\bar{u}}_j}{\partial x_i}\right) \end{aligned}$$

where ${\bar{S}}_{ij}$ is the strain rate tensor for the filtered field and $\delta _{ij}$ is the Kronecker delta. The WALE model variant for the eddy viscosity term, $\nu _{t}$, is given as:

5 $$\begin{aligned} \nu _{t} = (C_w \Delta )^2 \frac{\left( {\bar{S}}_{ij}^d {\bar{S}}_{ij}^d \right) ^{3/2}}{\left( {\bar{S}}_{ij} {\bar{S}}_{ij} \right) ^{5/2} + \left( {\bar{S}}_{ij}^d {\bar{S}}_{ij}^d \right) ^{5/4}} \end{aligned}$$

where $C_w$ is the constant of the WALE model, $\Delta$ represents the filter width and $\bar{S_{ij}^d}$is the traceless symmetric part of the square of the velocity gradient tensor.

Simulations are performed using the finite-volume solver pimpleFOAM in OpenFOAM. Fluid density and kinematic viscosity were set to 1060 kg m^−3^ and 3.78 $\times 10^{-6} \text{m}^2 \text{s}^{-1}$, respectively, and were chosen to be representative of blood. The simulation employs a implicit second-order backward scheme for temporal discretization, ensuring accuracy and stability in transient simulations. Spatially, a bounded second-order scheme was applied to limit nonphysical oscillations. The simulations converged at each time step to a residual of $1 \times 10^{-5}$ for velocity and pressure, ensuring numerical convergence of the governing equations. A variable time step was used which varied between $10^{-4}$ and $10^{-5}$, ensuring a mean Courant–Friedrichs–Lewy (CFL) number of less than one throughout the entire simulation.

### Analysis

#### Energy spectral density

To study the properties of turbulence, velocity data were collected near the coarctation site over 30 cycles. By applying a fast Fourier transform (FFT), this data can be converted to the frequency domain. With an approximate sampling frequency of 100 kHz, instantaneous data were gathered for the last 20 cycles. The energy spectral density $E(f)$ at frequency $f$ can be calculated using the following equation used by^77^

6 $$\begin{aligned} E(f) = \frac{1}{L} \left| {\mathcal {F}}(u(t)) \right| ^2 \end{aligned}$$

and ${\mathcal {F}}(u(t))$ is the Fast Fourier Transform (FFT) of the velocity signal $U(t)$. $L$ is the number of samples in the time-domain signal. $\left| {\mathcal {F}}(u(t)) \right| ^2$ represents the squared magnitude of the FFT, which provides the power at each frequency.

#### Phase-average method

**Fig. 9:**
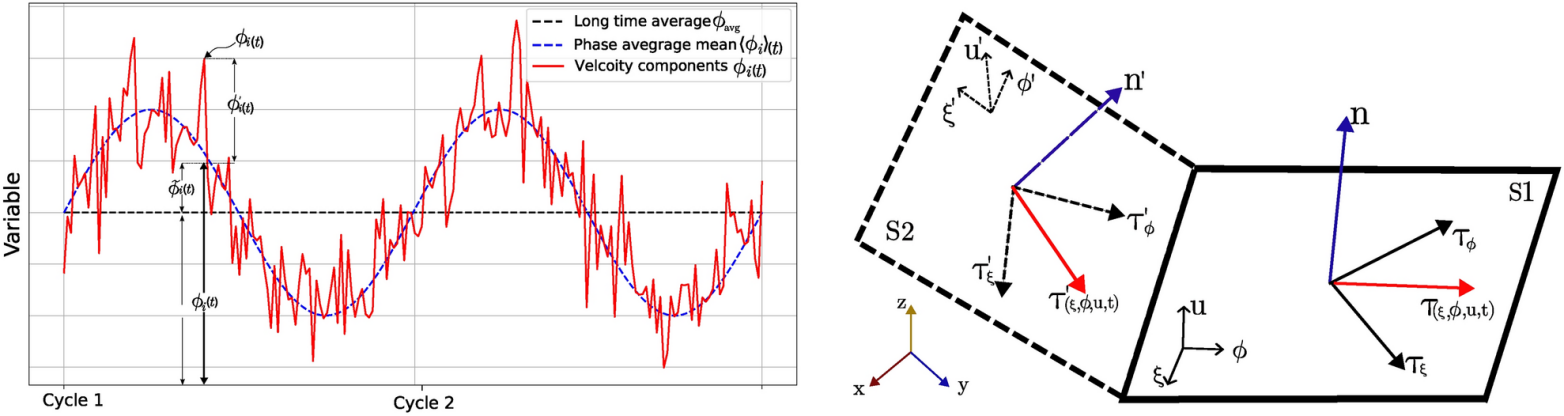
Triple decomposition (**A**) of a fluctuating variable in a periodic pulsatile turbulent flow. This variable’s time divides into a time-averaged part ${\bar{\phi }}(x)$, periodic pulsation ${\tilde{\phi }}_i(t)$, and turbulent fluctuation $\phi '_i(t)$. $\langle \phi _{i} \rangle (t)$ gives the phase-average value during the cardiac cycle. (**B**) Schematic of WSS tensor $\tau (\xi ,\phi ,u,t)$ on two mesh surfaces S1 and S2 in their coordinates.

To evaluate both mean and turbulence parameters in pulsatile flow, it is necessary to perform a decomposition. In periodic and pulsatile flow, the turbulence can be identified as the flow changes from one cycle to the next. This substantial variation between cycles is caused by the irregular and random motions of the turbulent structures within the flow. This process involves the decomposition of a given variable, $\phi _i$, into mean and fluctuating components as seen in Fig. 9A:

7 $$\begin{aligned} \phi _i(t) =\underbrace{\phi (x)_{avg} + {\tilde{\phi }}_i(t) }_{\langle \phi _{i} \rangle (t)}+ \phi '_i(t) = \langle \phi _{i} \rangle (t) + \phi _i'(t) \end{aligned}$$

where $\phi (x)_{avg}$ is the long time-averaged value, ${\tilde{\phi }}_i(t)$ is the periodic pulsation from inlet, $\phi '_i(t)$ is the turbulent-related resolved fluctuation. $\langle \phi _{i} \rangle (t)$ gives the mean value (phase-average) of a variable at a given time, *t*, during the cardiac cycle. This is defined as follows:

8 $$\begin{aligned} \langle \phi _i \rangle (t) = \frac{1}{N} \sum _{n=1}^{N} \phi _i(t+nT) \end{aligned}$$

where $T$ is the period of the cardiac cycle and $N$ is the maximum number of cycles over which averaging is performed from $n = 1$ to $n = N$. The fluctuation component is defined as the root mean square of the instantaneous and phase-average variables:

9 $$\begin{aligned} \langle {\phi _i'}(t)\rangle = \sqrt{\frac{1}{N} \sum _{n=1}^{N} (\phi _i(t+nT) - \langle \phi _i \rangle (t))^2} \end{aligned}$$

The mean and turbulent kinetic energies (KE and TKE, respectively) at a given time, $t$, are calculated as:

10 $$\begin{aligned} KE (t)= \frac{\rho }{2} \langle \phi _i \rangle (t) \cdot \langle \phi _i \rangle (t) \end{aligned}$$

11 $$\begin{aligned} TKE(t) = \frac{\rho }{2} \langle {\phi _i'}(t)\rangle \cdot \langle {\phi _i'}(t)\rangle \end{aligned}$$

Evaluating the proper convergence of the mean and statistical parameters is an iterative process. Consequently, we developed a four-phase data processing approach, as illustrated in the appendix. In summary, LES runs were performed on a predetermined number of cardiac cycles (phase 0); convergence was evaluated at systole peak by calculating the phase-averaged mean KE and phase-averaged TKE according to Eqs. 10 and 11; Simulations were determined to be converged when the difference relative to the previous cardiac cycle was less than 1% (phase 1). If convergence was not achieved, the simulation was set to run for additional cardiac cycles, and phases 0-1 are repeated until convergence is achieved. The phase averages of the full simulation were then calculated at all times exported within the cardiac cycle. To avoid transient effects, the initial cycle is discarded and phase averaging is performed using all other simulated cycles (phase 2). Finally, the parameters of interest are calculated using phase-averaged fields (phase 3). This method ensures convergence of both phase-averaged mean and phase-averaged turbulent properties, whilst minimising computational costs by utilising only the necessary number of cardiac cycles. In this study, between 20 and 30 cycles were needed to achieve the convergence $< 1\%$ of the phase-averaged KE and TKE. Phase-average graphs showing the convergence for each BPM are provided in the Supplementary Material.

#### Wall shear stress parameters

The primary force detected by the vascular surface, the endothelium, is the tangential WSS in the direction of flow, as shown in the Fig. 9B. To study WSS and evaluate its effects on the aortic wall, we employ several WSS metrics. Phase-averaged WSS (PAWSS) and turbulent WSS (TurWSS) are calculated using Eqs. 8, 9, and replacing $\phi _i$ with WSS. To understand the effects of blood flow on the arterial wall over several cardiac cycles, the time-averaged wall shear stress (TAWSS) is calculated by integrating PAWSS throughout the cardiac cycle. Furthermore, recognising the limitations of traditional metrics in capturing the multidirectional nature of blood flow, transverse WSS (TransWSS) is introduced. This metric complements existing ones by assessing changes in flow direction throughout the cardiac cycle. Lastly, the oscillatory shear index (OSI) is used to evaluate directional changes in the WSS by quantifying the deviation of the WSS vector from the TAWSS vector over a cycle. The following equations summarise all the WSS metrics and their corresponding equations^60^. To capture the periodic nature of the flow, all WSS and its indicators are computed from phase-averaged solutions as:

12 $$\begin{aligned} & WSS_i = \nu \left. \frac{\partial \langle u_i \rangle }{\partial y} \right| _{\text {wall}} \end{aligned}$$

13 $$\begin{aligned} & PAWSS = \frac{1}{N} \sum _{n=1}^{N} WSS_i(t_n + nT)\end{aligned}$$

14 $$\begin{aligned} & TurWSS = \frac{1}{N} \sum _{n=1}^{N} WSS_i'(t_n+nT), where \quad WSS_i'(t_n + \theta ) = WSS_i(t) - \langle WSS_i \rangle _{(\theta )} \end{aligned}$$

15 $$\begin{aligned} & TAWSS = \frac{1}{T} \int _0^T PAWSS dt \end{aligned}$$

16 $$\begin{aligned} & transWSS = \frac{1}{T} \int _{i=0}^{T} \left| \left( {\hat{\textbf{n}}} \times \left( \frac{\bar{\langle WSS_i \rangle }}{|\bar{\langle WSS_i \rangle }|} \right) \right) \cdot \langle WSS_i \rangle _{(nT)} \right| dt \end{aligned}$$

17 $$\begin{aligned} & OSI = \frac{1}{2}\left( 1 - \frac{|\bar{\langle WSS_i \rangle }|}{TAWSS}\right) \end{aligned}$$

## Supplementary Information

Supplementary Information 1.

Supplementary Information 2.

## Data Availability

The data sets used or analysed in this study are available from the corresponding author upon reasonable request. Study available from the corresponding author on reasonable request.
