# Supplementary material for: An in silico analysis of heart rate impact on wall shear stress hemodynamic parameters in aortic coarctation
Source: Sci Rep. 2025 Jan 22;15:2747. doi: 10.1038/s41598-025-85522-0 (PMC11751079; doi:10.1038/s41598-025-85522-0)
Supplement: Supplementary file 1 — Supplementary Information 1. [file 41598_2025_85522_MOESM1_ESM.pdf]

Supplemental Data

Phase-average Method

Evaluating the correct convergence of mean and statistical parameters is an iterative process. Consequently, we developed a four-phase data processing approach, as illustrated in Figure 1

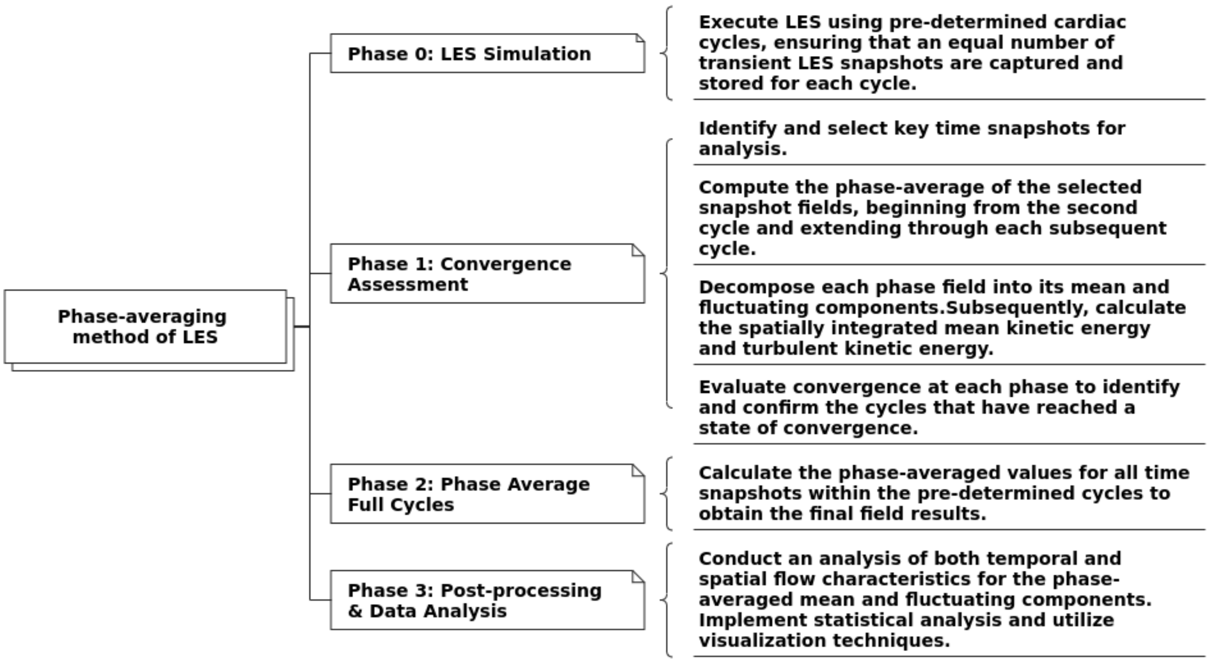

Figure 1. Workflow of Phase-Averaged Method

Numerical Convergence

The second half of the cycles was dedicated to evaluating different cardiovascular parameters in order to ensure that the findings reached accurate statistical convergence. This approach is commonly employed in cardiovascular studies to examine convergence[1,2]. Figure 2 displays the continuous phase-averaged KE and TKE across the aorta during peak systole. Furthermore, the continuous phase-averaged wall shear stress (PAWSS) and its fluctuation component, turbulent WSS (TurWSS) were calculated at selected surface points significantly influenced by jet flows. This methodology allows for the assessment of PAWSS and TurWSS throughout cardiac cycles, as demonstrated in Figure 3. Errors were calculated by comparing the last five cycles to the final cycle and are represented as error bars relative to the converged values. The results clearly demonstrate localised convergence, achieving errors of 0.01% for PAWSS and -2.63% for TurWSS. Due to the high sensitivity of WSS to both temporal and spatial variations, the 30-cycle phase-averaged method is appropriate for this study.

Mesh Sensitivity

A total of five unstructured meshes with approximately 2, 4, 6, 8, and 10 million cells were generated using the snappyHexMesh tool. Each mesh configuration featured five layers of prismatic cells near the wall. The final layer was designed to have a thickness equivalent to 20% of the size of the near-wall cell, with a growth rate of 1.1 between layers. To examine the sensitivity of the mesh, a study was performed considering peak systole flow conditions. Figure 4 indicates the locations where flow quantities are assessed. Figure 5 provides comparison of magnitude of mean velocity |U| and magnitude of its gradient of

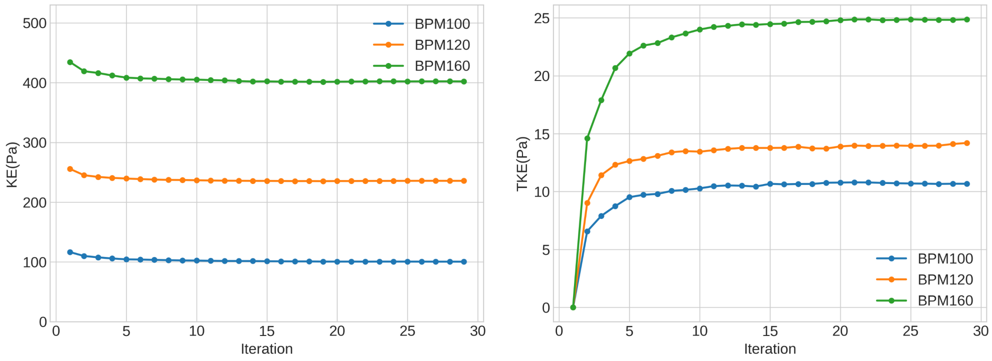

Figure 2. Executing a phase average on the mean KE and TKE, each integrated over the entire aorta at the peak systole activity

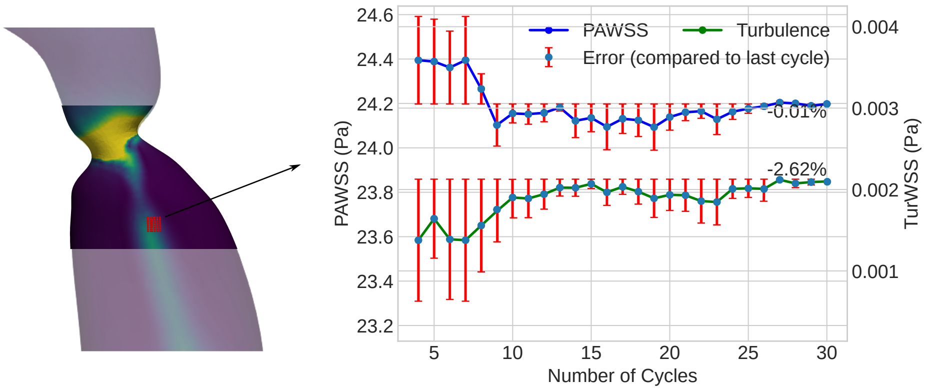

Figure 3. Executing a phase average on the PAWSS and TurWSS (Right), each integrated over highlight points(left) at the peak systole activity

the velocity vector |∇U| at four locations (Lines 1-4) while WSS is plotted along a circumferential slice downstream of the coarctation (Circle). As illustrated in the figure, lines 1 and 2 are located in regions of relatively stable flow, where minimal variation in both velocity magnitude and gradient across all mesh sizes. Convergence with progressive refinement is observed for Line 3 and 4, which is located in the region of interest (ROI); near the coarctation. For Line 3, M8 and M10 are similar but do not converge perfectly. A turbulence resolving simulation will not truly converge until all scales of motion are simulated, as in a Direct Numerical Simulation (DNS), which is deemed unnecessary here. The observed variance is expected to partly due to the requirement for longer time-averaging at this location, due to the greater range of scales of motion. The average deviation of mean WSS between the 8M and 10M meshes was 2.89% in the ROI, which is acceptable given the high sensitivity of WSS to spatial and temporal variations

To quantify the impact of local regions of the flow that were not perfectly converged, we also evaluated mesh convergence using global integrals over both the full domain and isolated regions of interest. Tables 1 and 2, compares region-averaged quantities of mean wall shear stress (MeanWSS), turbulent WSS (TurWSS), mean kinetic energy (KE) and turbulence kinetic energy (TKE). Table 1 provides the average over the entire domain, while Table 2 provides average quantities over a ROI centered on the jet flow impact area, as illustrated in Figure 4. Error values are recorded for all meshes, relative to values predicted by the 10M mesh. As shown, the 8M mesh returns maximum deviations of 2% across the entire geometry and 3.4% for the ROI.

Furthermore, we have assessed the ratio of resolved to total TKE for all meshes, a common metric used in Large Eddy Simulation to ascertain the quality of the simulation. We show that with the 8M mesh an average of 97% of TKE is resolved, while in the ROI this falls to 95%. For reference, the best practice generally recommends a value of 90% or more. Finally, the 8 million cell mesh maintains an average y-plus value below 1 and adequately covers 99% of the vessel wall surface, ensuring accurate shear stress computations post-coarctation.

In conclusion, this mesh sensitivity study concludes that the 8M mesh is sufficient for accurately capturing the flow dynamics in the aorta with CoA. While minor deviations between the 8M and 10M meshes were observed in the descending aorta, the convergence of region-averaged quantities such as WSS, KE, and TKE – as well as the high percentage of resolved TKE – combine to demonstrate a satisfactory level of mesh refinement for our purposes. The 8M mesh effectively captures the complex flow features and turbulent structures critical for accurate hemodynamic analysis.

	Mesh Size	2M	4M	6M	8M	10M
Percentage error relative to 10M	MeanWSS	15.6%	9.44%	3.11%	1.54%	-
	TurWSS	8.55%	4.21%	3.73%	2.15%	-
	KE	3.88%	2.12%	1.04%	0.7%	-
	TKE	5.41%	2.9%	1.54%	0.52%	-
	SGS model contribution to TKE	8.57%	7.1%	5.08%	3.15%	1.2%

Table 1. Mesh characteristics and mesh sensitivity results over entire aortic geometry.

	Mesh Size	2M	4M	6M	8M	10M
Percentage	MeanWSS	20.09%	12.45%	6.14%	2.89%	-
error	TurWSS	12.75%	7.43%	4.77%	3.4%	-
relative to	KE	7.94%	4.87%	3.59%	1.34%	-
10M	TKE	8.11%	5.76%	4.04%	0.79%	-
	SGS model contribution to TKE	15.97%	9.15%	7.01%	4.63%	3.37%

Table 2. Mesh characteristics and mesh sensitivity results over the isolated region of coarctation and descending aorta.

Three-element Windkessl Model Coefficient

The 3EWK coefficient in the outlet boundary conditions used in this study is presented in the table below.

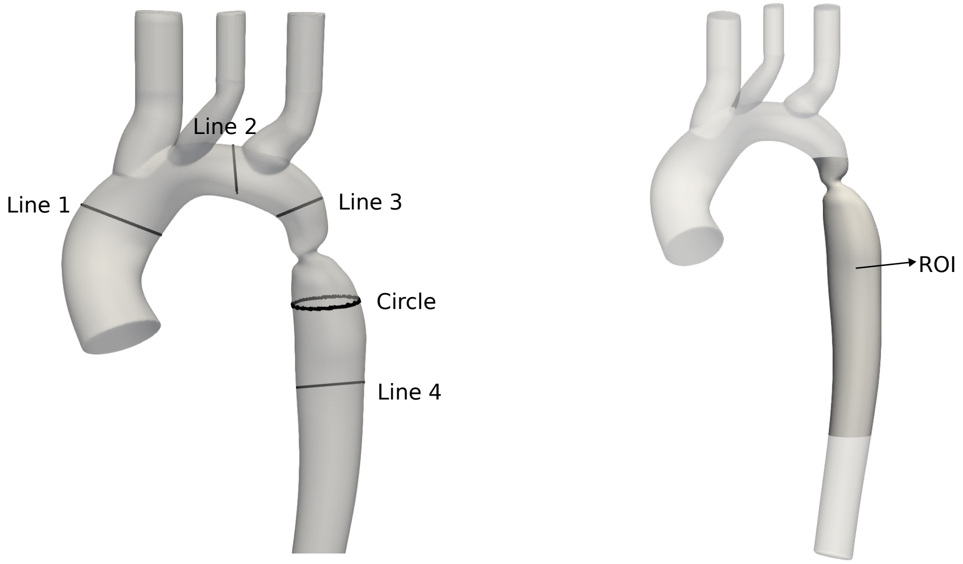

Figure 4. Ilustration of the selected cross-sectional locations for mean velocity analysis (right); The region of interest (ROI) for quantitative analysis—highlighting the jet flow impact area, including the coarctation and descending aorta (left).

Table 3. Outlet 1 - Brachiocephalic Artery

[h!p]

Coefficient	BPM100	BPM120	BPM160
Z($Pa \cdot s \cdot m^3$)	2.8518e8	2.8518e8	2.8518e8
R($Pa \cdot s \cdot m^3$)	1.2716e9	1.0121e9	6.8781e8
C($m^3 Pa^-1$)	1.2333e-9	1.4800e-9	1.9733e-9

Table 4. Outlet 2 - Left Common Carotid Artery

Coefficient	BPM100	BPM120	BPM160
Z($Pa \cdot s \cdot m^3$)	8.7265e8	8.7265e8	8.7265e8
R($Pa \cdot s \cdot m^3$)	3.2445e9	2.5583e9	1.7005e9
C($m^3 Pa^-1$)	4.6635e-10	5.5962e-10	7.4615e-10

Table 5. Outlet 3 - Left Subclavian Artery

Coefficient	BPM100	BPM120	BPM160
Z($Pa \cdot s \cdot m^3$)	4.8131e8	4.8131e8	4.8131e8
R($Pa \cdot s \cdot m^3$)	1.9727e9	1.5637e9	1.0525e9
C($m^3 Pa^-1$)	7.8238e-10	9.8836e-10	1.2518e-9

Table 6. Outlet 4 - Descending aorta

Coefficient	BPM100	BPM120	BPM160
Z($Pa \cdot s \cdot m^3$)	1.9865e8	1.9865e8	1.9865e8
R($Pa \cdot s \cdot m^3$)	1.7905e9	1.4590e9	1.0446e9
C($m^3 Pa^-1$)	9.6524e-10	1.1583e-9	1.5444e-9

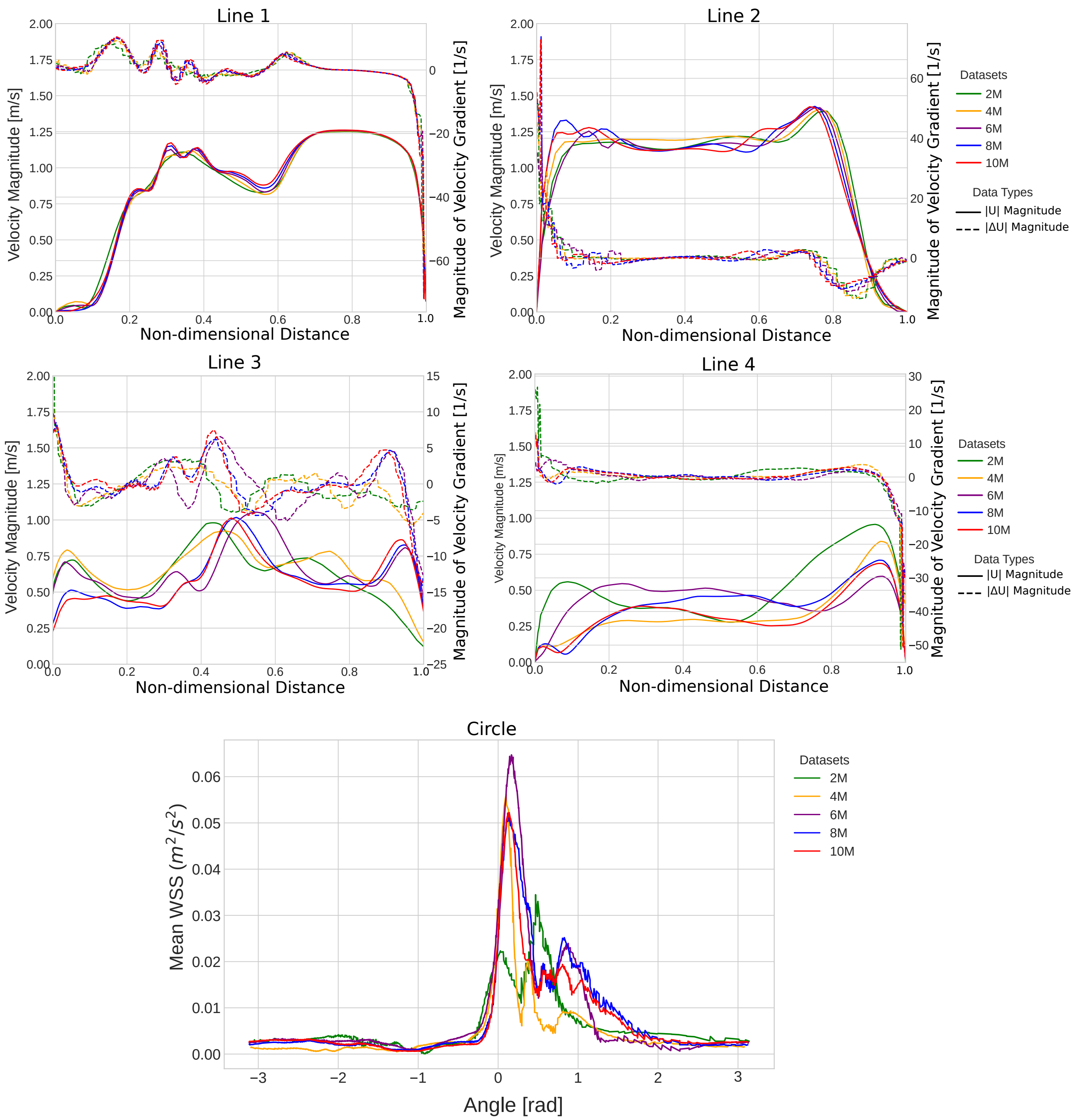

Figure 5. Magnitude of mean velocity vector and the magnitude of the gradient of mean velocity vector plot of mean axial velocity at 4 cross-sections with varying mesh sizes based.

References

1. Lantz, J., Gårdhagen, R. & Karlsson, M. Quantifying turbulent wall shear stress in a subject specific human aorta using large eddy simulation. *Med. engineering & physics* **34**, 1139–1148 (2012).

2. Manchester, E. L. *et al.* Analysis of turbulence effects in a patient-specific aorta with aortic valve stenosis. *Cardiovasc. engineering technology* **12**, 438–453 (2021).
